# “Ok—I Need Help from Somewhere”: ‘The Educational Value of Multiplex Student Relationships in a Commuter College’

**DOI:** 10.1007/s10755-022-09611-y

**Published:** 2022-06-09

**Authors:** Annika Fjelkner-Pihl

**Affiliations:** Center for Higher Education Development, Kristianstad University, Kristianstad, Sweden

**Keywords:** Commuter students, Commuter college, Mixed methodology, Social network, Multiplex relations

## Abstract

The present mixed-methods study provides insight into how students in higher education describe and form multiplex relationships in a cohort of students attending a commuter college, thereby improving our understanding of the complex relationships within student groups and their relation to learning. The main aim was to understand the student experience of networking with other students, particularly how commuter students perceive their academic *multiplex* relationships. Relational data were collected in a cohort of students (*n* = 109), complemented by 15 semi-structured interviews. One main finding was that students perceived that their largely homophilous multiplex relationships were central to academic achievement, but if students also had limited friendship relations these multiplex relationships could limit students’ academic experience. Another finding was how orientation week and group work done during the first semester mainly supported the formation of multiplex networks but were also perceived as barriers by some students. Likewise, commuting both scaffolded network building and became a barrier, especially for students with an immigrant background. One important implication for curriculum development is that faculty cannot leave relationship building to the students alone. A strategic model is discussed that supports emerging multiplex relationships, which can lead to gains in learning, retention, and integration.

Students generally perform better in relationship-rich environments, and the classroom is a central place for the formation of supportive relationships (Felten & Lambert, 2020). They also benefit from interaction with faculty and peers, while orienting themselves in courses (Esterhazy, 2019). These interactions shape the students’ experience of their education, of themselves as learners, and perhaps even how they view the world (Henderson et al., 2019). Thus, it is useful to reflect on how we organize higher education in the future, particularly now in the wake of the emergency transition to online learning during the Covid-19 pandemic. As students’ opportunities to interact with peers reduced drastically, face-to-face interaction with peers decreased, leading to negative effects on their self-reported well-being (e.g., Elmer et al., 2020; Wang et al., 2020/2021), but also to decreased motivation, and problems related to structuring information about courses and dealing with feedback (Warfvinge et al., 2021). Hence, it is particularly important to understand how students find support in each other as they strive to orient themselves in higher education, whether in online or face-to-face learning environments.

Students maintain various types of relationships with their peers, for example working, learning or friendship relations (e.gHommes et al., 2012, 2014; Rienties & Tempelaar, 2018; Rienties et al., 2012). Working and learning relationships provide task-related support (Rienties & Tempelaar, 2018), whereas friendship relations provide emotional support and access to information, both of which are important for academic achievement (e.g., Hommes et al., 2012). Previous research has mainly considered these types of relationships in isolation (e.g., Biancani & McFarland, 2013); this perspective, however, may be overly simplistic, as student relationships are often multi-layered, that is, multiplex in nature. A relationship is considered multiplex when, for example, students are both friends and working partners (e.g., McCabe, 2016).

How students perceive, form and maintain multiplex relationships in educational settings, especially where a large share of students commute, has been underexplored. The substantial overlap between relationships reported in previous research (e.g., Chen et al., 2012; Author, 2021; McCabe, 2016), and the importance of multiplex academic relationships discussed by McCabe (2016) and Felten and Lambert (2020), indicate the relevance of exploring student multiplex relationships further, especially given that, during the pandemic, multiplex relationships were found to be more resilient (Elmer et al., 2020). Commuter students are of specific interest because they maintain pre-college friendship relations to a greater extent and are less engaged in campus activities (Alfano & Eduljee, 2013). As their pre-college friendships offer social support, commuter students may be less inclined to form new social relations, which in turn may limit their possibility to form academically supportive relations at university.

The main aim of the present study was to try to understand how commuter college students form and perceive their multiplex relationships and the support they offer, thereby improving our understanding of the complex, intertangled relationships existing within student groups, and in addition possibly providing an alternative image of commuter students. The results and implications of the study may be of interest to management, academic developers and staff engaged in teaching, student support, or planning of higher education, as well as other researchers who study students’ social relationships.

## Literature Review

Because the present aim was to explore how commuter college students form and perceive their multiplex relationships and the support they offer, to provide context, the literature review will first discuss support offered by student social networks generally, and then the specific circumstances of commuter students and the barriers they may face.

### Research on Student Social Networks

Students’ study-related networks provide emotional and social support, but also information and cognitive processing support (Tomás-Miquel et al., 2016). Social Network Analysis (SNA) studies typically focus on groups formed during one module or semester, that is, learning communities of groups of 10–15 students formed by faculty for a specific reason (Brouwer & Jansen, 2019). In the present study, the groups (or networks) are informal peer networks formed by students themselves during two years of study.

Network studies in higher education have explored various types of relationships separately (uniplex relations), commonly student working, learning or friendship relations (e.gHommes et al., 2012, 2014; Rienties & Tempelaar, 2018; Rienties et al., 2012). The relationships studied are often described as either instrumental or expressive. Working and learning relationships are normally considered *instrumental*, in that they arise due to a work role (Methot et al., 2016) or, in this case, an assigned group. Such studies have explored with whom students communicate, both formally and informally, about task-related activities (Rienties & Tempelaar, 2018), such as how to solve an assignment or how or what to study for an examination. It is common for students to maintain fewer working and learning relationships than friendship relations (Author, 2021; Rienties & Tempelaar, 2018).

Friendship networks, on the other hand, are based on *expressive relationships* which involve passive information diffusion (Hommes et al., 2012). Expressive ties are not bound to any formal structure, such as assigned groups or learning communities in a classroom situation, instead they are based on voluntary interaction (Methot & Lepine, 2016). Rienties and Tempelaar (2018) pointed to how the information sharing that takes place in friendship relations, outside the formal work group, is important in that it helps students avoid groupthink and achieve creative solutions to group assignments.

However, students often share several types of relationships, which means that these relationships are multiplex, rather than separate independent constructs (uniplex). Multiplex relationships, being simultaneously both expressive and instrumental, are rewarding for students; they contribute intellectual engagement, inspiration, and emotional and instrumental support, all of which are success factors in college (Felten & Lambert, 2020; McCabe, 2016).

In general, students, like people in society at large (e.g., McPherson et al., 2001), tend to form friendship relations with students they perceive are like themselves (*homophily*), or have easy access to (*propinquity*), that is, with students in the same courses or cohort/study program (e.g., Gašević et al., 2013; Hommes, et al., 2014). Likewise, in a study on social integration and social support, Wilcox et al. (2005) concluded that making compatible friends in college is important for social integration and that student living arrangements are vital for this process. In a study on students’ social networks in an American university, McCabe (2016) found that students mainly met their new college friends in various communities outside class, such as fraternities or sororities, campus clubs and other activities, while also maintaining a network of old friends from home.

### Commuter Students and Study-Related Relations

The students in the present study commute to a large extent, which means that the classroom and the commute itself potentially affect how students form relationships and their academic social network. There is much research focused on how commuter students are disadvantaged and face several barriers to participation. For example, previous research has indicated that commuter students are often older (25 +), come from ethnic and/or socially disadvantaged backgrounds, and are often first-generation students (e.g., Alfano & Eduljee, 2013; Newbold, 2015). Furthermore, they are less likely than non-commuter students to fully engage with their peers or feel they fit in (e.g., Pokorny et al., 2017). Commuter students are also more likely to leave campus immediately after class, and they less frequently attend social activities on campus due to other engagements, such as work or family obligations (Biddix, 2015). For this reason, it is also more difficult for them to form study-related relationships.

Commuting has further been found to have a negative impact on engagement, and time spent commuting negatively affects academic outcomes (e.g., Author, 2021; London Higher, 2019). Students from disadvantaged backgrounds are most likely to commute, whereas privileged students most often live on or close to campus (London Higher, 2019).

However, it must be noted that as more students commute, this somewhat simplified picture of commuter students as a struggling and disadvantaged group may no longer hold true. Research linking commuting to academic outcomes is inconclusive. Gianoutsos and Rosser (2014) found no difference in retention and academic standing, and other studies have further suggested that there is a substantial overlap between commuting and other factors, such as ethnicity, that explain the attainment gap (Author, 2020). In addition, a qualitative pilot project indicated that students also found commuting and living off campus advantageous. For example, they found friends during the commute and tried to use travel time effectively. They also seemed to treat studying as a full-time job, dividing their time between study (work) and leisure time for socializing with friends and family at home (Thomas, 2019).

In sum, although the situation of commuter students is complex and diverse, they do seem to face several, overlapping barriers to participation that may affect how they form and maintain multiplex study-related relationships, which in turn may affect academic outcomes. Universities could do more to counteract the barriers faced by commuter students, which is especially important as more and more students commute. The literature commonly refers to three types of barriers: institutional, situational, and dispositional (e.g., Goto & Martin, 2009). Institutional barriers involve policies and practices of the higher education institution (e.g., Goto & Martin, 2009), such as tuition rates or the cost of course literature, access to information or inconvenient course times. Policies and practices of higher education institutions are often shaped after the needs of campus students, the image of which has remained an ideal type of what it means to be a student. These are also possible for universities to modify to better suit the needs of commuter students. Situational barriers concern the circumstances of the individual, such as health issues or availability of childcare (Patterson, 2018). Dispositional factors include the student’s attitude and self-perception, self-esteem, self-efficacy, and motivation (Patterson, 2018).

### Research Questions

Given the special situation of commuter students and commuter colleges, it is of interest to explore the multiplex character of student relationships in that specific setting. The overarching aim of the present study was to explore how students in a commuter college perceive and form multiplex relationships and what potential implications their experiences have for the organization and planning of the program. Use of a mixed-methods approach enabled a more contextualized analysis of the cohort in question, providing qualitative information that will allow us to better understand students’ experience of the mapped relationships.

1. How do commuter college students describe their study-related multiplex relationships?
2. How do commuter college students form study-related multiplex relationships?

## Study Context

This two-step study targeted a cohort of business students at a teaching-intensive university in Sweden, with about 14,000 students predominantly enrolled in teacher education, nursing, and business programs. The business program enrolls about 600 students in total, mainly at the bachelor’s level. It is important to note that 70% of the students in this cohort commuted more than two hours each day and that 35% had an immigrant background, that is, they were either born abroad or both parents were born abroad (Swedish Higher Education Authority, 2019). Only 30% of the students had two parents with an academic degree. The study was part of an evaluation of the program, as faculty had previously pointed out issues with integration and completion rates. I have taught various modules in the program for the past ten years and have been intrigued by group formation patterns, and the variation in integration and completion rates among the students. I was not active in teaching or grading when the study was being conducted.

Students are divided into classes of 30–70 students that remain stable throughout the full three-year program. Most courses are compulsory, and students are required to take them in a specific order. Thus, the context differs substantially from that of most research on student social networks where students instead have more educational choice, live on campus to a greater extent, and form relationships in dormitories, extra-curricular activities on campus or in organized learning communities.

The opportunities for forming relationships were many during the first semester. In the first week of the first semester, the Student Union arranged orientation activities, and students were given several group assignments to be performed in different group constellations. Students were purposely divided into work teams based on variation in gender, language background (native/immigrant) and place of residence (commuter/on campus), the goal being to enable them to form study-related relationships. However, after the first course, during which most of the orientation activities took place, students were mainly free to form their own groups.

## Method

The present mixed-methods social network study is explorative (Cohen et al., 2007), as I attempted to gain insight into the phenomenon in focus rather than to draw general conclusions. Prior social network studies have focused on uniplex friendship, working, or learning relationships, respectively, rather than multiplex ones. Definitions of network relationships vary across studies (Rienties & Tempelaar, 2018). Arguably, the context of a commuter college in a Swedish university setting differs from the context of most prior research. This calls for an explorative interpretivist approach, which I chose, and which allowed me to focus on the participants’ own descriptions of the relationships under study and how they are formed. My aim was to try to understand the phenomenon from the participants’ perspective, to the extent that this is possible (Braun & Clarke, 2006).

### Procedure & Analysis

#### Social Networks

In the first step of the study, students completed a questionnaire exploring their working, learning and friendship relations using a standard closed-network roster technique (e.g., Heliot et al., 2019; Rienties & Tempelaar, 2018). Participants answered three questions, requiring them to mark students in their respective specialization whom they “work a lot with,” “are friends with” and “have learned from.” The survey was distributed during a lecture. Students were informed that participation was voluntary, and that data handling would be done so as to ensure anonymity and confidentiality.

Networks were represented using Netdraw, in UCINET. Centrality measures were calculated using the software UCINET v. 6, a program developed for social network analysis (Borgatti et al., 2002). Freeman’s in-degree centrality was used to measure centrality for the *working/ learning/friendship networks* (Grunspan et al., 2014).

*Independent-samples t-tests* were performed in SPSS (IBM Corp. Released 2016. IBM SPSS Statistics for Windows, Version 24.0. IBM Corp.) to test for group-level differences in number of relations. Differences were considered statistically significant if *p* < 0.05, two-tailed (Table 1).

**Table 1:** Sample questions from the interview guide

Category	Main questions	Sample follow-up question
Warm-up question	What does it mean to be a student?	
Network questions – with each student’s Social Network questionnaire as a discussion point	Whom have you marked that you work a lot with? Whom have marked that you have learned from? Whom have you marked as friends?	Why them? How did you get to know them? Could you give examples of how you work/hang out? In what way is your relationship with them different from the guys you work a lot with?
Closing question	If you think about your school network, in what way has that network affected you, your studies/learning?	

*Note.* The exact wording of the questions varied depending on how the conversation with the students went.

**Table 2:** T-result comparing network scores between non-commuters and commuters for all, immigrant, and Swedish students

*Sample*	*Variable*	*Predictor*	*M (SD)*	*Range*	*t* (df)	*p*
All	Working NW	Swedish	2.82 (1.57)	0–8	5.07 (144)	0.000
		Immigrantͣ	1.50 (1.41)	0–5		
	Friendship NW	Swedish	7.62 (4.05)	0–17	3.57 (144)	0.000
		Immigrant	4.94 (4.87)	0–20		
	Learning NW	Swedish	3.52 (2.31)	0–10	5.57 (144)	0.001
		Immigrant	1.57 (1.45)	0–5		
	MPX NW	Swedish	2.03 (1.36)	0–7	5.30 (144)	0.000
		Immigrant	0.89 (1.06)	0–3		
Swedish students	Working NW	Non-commuters	2,77 (1.44)	0–5	0.59 (81)	ns
		Commuters	2.98 (1.60)	0–8		
	Friendship NW	Non-commuters	7.90 (3.34)	0–20	0.44 (81)	ns
		Commuters	7.94 (4.18)	0–17		
	Learning NW	Non-commuters	3.68 (1.96)	0–5	0.17 (81)	ns
		Commuters	3.75 (2.51)	0–10		
	MPX NW	Non-commuters	1.98 (1.14)	0–3	0.93 (81)	ns
		Commuters	2.25 (1.44)			
Immigrant students	Working NW	Non-commuters	2.86 (1.57)	0–5	-2.75 (36)	.009
		Commuters	1.42 (1.18)	0–8		
	Friendship NW	Non-commuters	9.57 (6,08)	0–20	-2.18 (36)	.036
		Commuters	5.10 (4.65)	0–17		
	Learning NW	Non-commuters	2.14 (0.90)	0–5	-0.65 (36)	ns
		Commuters	1.74 (1.57)	0–10		
	MPX NW	Non-commuters	1.00 (1.00)	0–3	-0.71 (36)	ns
		Commuters	1.03 (1.11)			

#### Semi-Structured Interviews

Respondents for the semi-structured interviews were selected based on *sequential purposeful sampling* (Palinkas et al., 2015) from the 25 students who had indicated their interest in being interviewed in the initial SNA survey (*n* = 109). Fifteen students were selected based on their profiles for the sample, the aim being to reflect as well as possible the composition of the cohort regarding, gender, and background (i.e., commuter/campus students, native/immigrant background) and at the same time have an equal number of students with small, medium, and larger sized friendship networks (see Table 3).

**Table 3:** Demographic information on interview participants

ID**	Gender	Age	Immigrant Background	Achievement*	Interview Time (min)	Commute (hrs)	Friends NW**	Mpx NW
Stella	F	22	Native	Medium	31	2	5	1
Taylor	F	22	Immigrant	Medium	30	2	3	1
June	F	25	Native	High	42	3	4	1
Monawar	M	23	Immigrant	Low**	45	3	2	2
Ahmed	M	23	Immigrant	Low**	37	3	3	1
Lottie	F	23	Native	Medium	47	0	12	3
Frank	M	30	Native	Medium	28	0	8	5
Penny	F	24	Native	High	46	0.75	6	1
Tara	F	24	Native	High	42	2	6	1
Inez	F	22	Immigrant	Low	49	3	8	4
Jamie	M	25	Native	Medium	42	0	20	3
Carl	M	28	Native	Medium	60	0	21	3
Ed	M	28	Native	Medium	43	1.5	20	3
Mina	F	26	Native	High	43	0	24	9
Mary	F	26	Native	High	37	2	17	2

67% commuters (68%), 60% female students (56%), 73% native background (63%). Composition of cohort within parentheses. * Achievement is calculated as share of courses in which the highest grade was achieved (Pass with distinction). In this specific program, the course grades awarded are Fail, Pass and Pass with distinction. High =  > 70%; Low = 10-69%; Low =  < 10%, as Grade point average (GPA) is not a standard calculation in the Swedish higher education system. ** 1-2 years extra completion time. **Student names are fictive

The aim was met regarding share of commuters and spread in network size (friendship), which was important as these two aspects are the focus of the study. There was also a mix regarding academic achievement, with three students categorized as low, five as high and seven as medium achievers. The fact that there were slightly more female and native Swedish students in the sample was deemed acceptable. The small N and the uniqueness of the case preclude drawing any general conclusions, but rather offer propositions (Crouch & McKenzie, 2006) that might give valuable insights into how students form and maintain multiplex study-related relationships that may be relevant to others in similar higher education contexts.

During the interviews, the paper SNA survey was used as a discussion point to help the students focus on both structural and compositional information on their networks. Table 1 presents sample questions.

Students were informed of the aim of the interview, and that they could end the interview at any point without giving an explanation. They were further ensured confidentiality in the handling and presentation of the data. All participants signed a consent form before the interview took place, in line with ethical the university’s guidelines.

The interviews were recorded and transcribed verbatim, whereafter they were analyzed using an inductive thematic approach (Braun & Clarke, 2006). The analysis was data driven, and initial codes – based on, for example, recurring words – were grouped together to generate themes. One theme was *Meeting spaces*, with subcodes such as “first group work” or “commute” (see Table 4 in Results). Identified themes were then discussed with experienced colleagues outside the project, who independently judged the themes prior to the discussions.

**Fig. 1:**
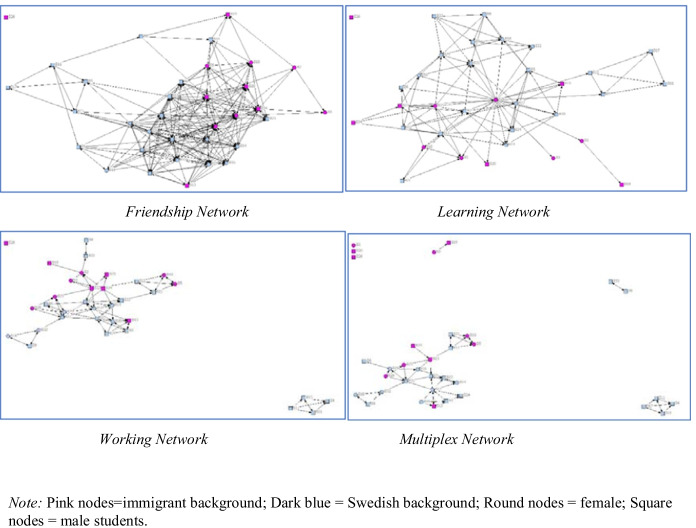
Sample group level friendship and learning networks

**Table 4:** Theme “Meeting spaces.” Subthemes and examples

Subthemes	Examples of meaning units
First group work & from commuting	It was them I got to know first. In the first and second course. For example, J lived in X [city] too. We got to know each other on the train. […] then it was S who was friends with W, so we started hanging out. (Inez)
First group work & old acquaintances	I mainly hang out with Jamie, and G. […]. I knew R a bit from before. He and I come from the same place, and he was going out with the sister of a friend of mine. Then we ended up in the same group in the first course and G was also in that group. (Frank)
From high school, propinquity	M and I, we went to the same high school as S. We usually work at home, or we also sit in building X. We study in X [city] as we all live in X. […]. (Monawar)
Orientation week	I have to say that orientation week is important to go to. It’s no joke. /…/ I feel as if not that many have quit because all my friends are still here, but I think that many, those who didn’t participate /…/ they were directly a bit on the outside. (Penny)

## Results

### The Networks

Visualization of the relational data provided insight into the complexity of relationship building in a cohort of students (N = 146; *n* = 109; response rate 75%). Students maintained several relations with other students with whom they discussed study-related issues (Fig. 1). On average, students had 7 friendship relations, had learned from 2.8 people, and worked closely with 2.4 people. Some had very large friendship networks, whereas others were highly selective and said they interacted with only a few people on a regular basis.

Students reported having multiplex relations with only a few students (2.7 relations on average). The color coding and shapes of the symbols in Fig. 1 indicate that these networks were largely based on homophily regarding gender and nationality. For example, immigrant female students (pink circles) tended to work mainly with other immigrant female students, and male Swedish students tended to work with other male Swedish students (blue squares). The multiplex and working networks were also more fragmented than the denser friendship network.

Immigrant students had significantly fewer relations than Swedish students did, on average. There were significant differences between commuters and non-commuters only among immigrant students, where commuter immigrant students had approximately 50% fewer working and friendship relations compared to non-commuting immigrant students. There was no significant difference between commuting and non-commuting Swedish students (Table 2).

#### Students Interviewed

The participating students were representative of the student population in the larger cohort regarding immigrant background and time spent commuting (see Author, 2020). In Table 3, the interviewed students are divided into three groups based on number of friends. Students in the bottom category with the most friends (16 +) also had larger multiplex networks overall. These were all native Swedish non-commuters who were older than 25. Students in the first category with few friends also had few multiplex relations. They also tended to commute longer distances, 2–3 h. Three out of five students in this category were immigrants, and two were struggling academically.

Two students with 6–15 friends also reported having only one multiplex relation, whereas the other students in that category had 3–5 multiplex relations. Only one student in this group had an immigrant background, and there was a mix between commuters and non-commuters.

#### How do Commuter College Students form Their Multiplex Networks?

The interviews revealed that the students’ multiplex relations were central to them, and it was there their main learning and work took place. Visualization of the networks also indicated a close overlap mainly between the multiplex and working networks, but also the learning network (Fig. 1).

Students pointed to four main ways in which they had gotten to know students they formed multiplex relations with: group work in the first semester, orientation week, old friends from high school, and fellow commuter students (Table 4).

Mary was a high-performing and well-connected student with 17 peers in her friendship network, but only two multiplex relations. She reported the two ways in which she met her two multiplex relations. *L is the one I’ve known since the first day […] we were placed together in the two first assignments.”* and “*A, we met on the train*.” She also explained how students formed friendship ties during *orientation week*: “I *was there […] yes, sense of community and such. We became pretty close those who were there.”*

Inez also talked about how she met one of her friends when they commuted to class. They later formed a multiplex network of four immigrant girls who all commuted from the same area. She explained how that made it easier to meet up and study together without having to commute to school:

[When we talk on the train] it’s not that often about that [school]. It’s when something has been difficult, then we can sit and discuss. When we had computer lab it was good to discuss, ‘did you understand this in this way’ and talk about it so that you could do it correctly later.

Here, Inez explained how she used the commute mainly as a social space but also a workspace, which was an experience she shared with other interviewed commuter students.

Another way students formed multiplex relations was with people they already knew from high school. Stella explained “*I knew her from the start and then it’s easy to stick together*.” Taylor had a similar experience:

In the beginning then I stayed with S and then you saw during the first month how groups were formed and then I was not in those groups. It was safe, as you saw others who didn’t know anyone.

Old friendships created safe havens, but then seemed to prevent students from connecting with other students later. The two immigrant male students in the sample told a similar tale. They found it difficult to get to know other students. According to Monawar, it was especially difficult to hang out with Swedish students; he said, “*you know orientation week is not for us*.” He referred to how some students with an immigrant background refrain from participating for religious or cultural reasons, or simply because they feel they do not belong. Typically, the student union runs orientation week, which involves games and other social activities, but also partying and alcohol consumption. None of the immigrant students in the sample had participated in orientation week.

All students in the sample mentioned the random assignment to groups for the group assignments during the first semester, like Jamie who concluded that “they ended up in the same group.” Many related that this was how they had met their multiplex relationships. Carl explained:

It was the first [group] work /…/ and then we were just put together /…/ and then this girl also thought it was important so we said, we will fix this /…/ and we have worked together ever since.

Even those students who had a bad experience of group work during the first semester found it important to at least have someone to sit with when they came to class, although they did not remain friends afterwards.

#### With Whom do Students Form Multiplex Relationships?

The interviewed students found the multiplex networks central, as it was here the actual academic work was done. Students learned the ropes from their multiplex relationships; they learned how to study and to write better.

When students talked about why they formed multiplex relationships with certain people, they said they felt similar to them in some respect, that is, had the same level of ambition, worked in the same way, or complemented each other. In line with previous research, the students formed networks based on homophily (e.g., Rienties & Tempelaar, 2018). Tara, for example, said she first got to know a group of girls during orientation week; she explained:

I don’t know if it’s age-related. She’s older, we’re both older. All the people I hang out with are the same age. I don’t know if that’s why… We didn’t know each other [from before].

Similarly, other students said they worked closely only with other commuters or only with other locals. Their stories indicated that their networks were based on both *homophily* and *propinquity*.

The results showed that student multiplex networks were highly divided by native background and gender (Author, 2020), which means that social homophily was a very strong determinant of the formation of multiplex relationships. This was evident despite the efforts made by the program to mix students randomly in groups during the first semester so that they could forge friendships. Monawar found it difficult to hang out with Swedish students. Jamie camouflaged the sensitive issue and said: “There is a clear divide between those who commute and people [from here].” The quotation indicated the strength of homophily and the resulting divide between immigrant and Swedish students, disguised by the fact that a large share of immigrant students were commuters.

Another reason students chose to continue working together was that they found they “*worked in a similar way*.” Stella elaborated:

We know how we work best together. […]. We’re very much alike. We both work the same way. Both like to plan, to be done with things a few days before deadline and avoid stress. We work alike

What Stella focused on in the quotation was rather the *work process*, or how they planned and organized the group work. Several of the students were *process oriented* and said that they wanted to do work in time and avoid stress, mentioning how important having the same attitude was to avoiding conflict with their co-worker.

Students also formed multiplex academic relationships with other students for a more goal-oriented reason: They chose other students they perceived had the *same level of ambition* as themselves. Ed was both process and goal oriented, and he described why he preferred working within his multiplex network:

We’ve worked in other groups along the way, but then we haven’t been satisfied with how things worked or the level of ambition. You know, good planning and that things get done, and if there’s a problem you ask each other.

It is worth noting here that the interviews took place during the fifth semester. Thus, all students were high achieving in the sense that they had been allowed to continue to the final year. However, several of them barely passed examinations, whereas others in the sample had high grades. In addition, there was some difference in the number of credits they had earned. It is evident that the level of ambition differed, from barely pass to the highest grade. Despite these differences, the same explanation regarding level of ambition was used by both high- and low-achieving students alike.

Although students said they formed networks based on similarities, they also frequently described how they had multiplex academic relationships with students because they complemented each other. These students were conscious of how they drew on each other’s strengths. For example, June, Inez, Ahmed, Carl, Ed and Tara all mentioned how they benefitted from working with their multiplex relationships because they *contributed different things*. Carl described how he had gotten to know Lottie:

If anything, I’m good at taking responsibility and there was this girl in the group, I don’t know why, but who also thought it was important. […] She’s academic and knows how to write and I’m more mathematical, and we’ve studied together since then and that’s why we do well. I’ve learned how to write in a way I didn’t know was possible […] and I’ve taught her how to study for exams.

In this quotation, Carl noted a similar level of ambition, but also how their collective strengths helped them achieve their goals, even indicating that he felt he would never have been able to achieve the same result without what he had learned from her.

Tara elaborated on the same topic:

They give me what I need so to speak. […] L is more like me, in a way and we both want to get things done fast, thoroughly, it should be on time, it must be planned and if you don’t understand you find out why. […] Sometimes you want something else, and Penny and I are a bit more opposites, but we complement each other pretty well. What she doesn’t think of, I think of and vice versa.

Penny said she had actively befriended Tara. She realized she needed help with structure, study skills and motivation to succeed. Penny was aware that she contributed language skills and creativity. June, likewise, worked closely with a girl who constantly failed exams on the first, and even second try. However, she was a much better writer, according to June, and when they worked together on group assignments their collective strengths enabled them to achieve the highest grade most of the time. Students referred to how they liked working closely with their multiplex network because they trusted each other. A high level of trust is one key aspect of multiplex relationships. Ed, for example, explained how “*There is high trust and that I think is important when you work together and a high level of ambition.”*

## Discussion & Implications

The present study’s contributions to the literature are insights into how students describe and form multiplex relationships; the study also improves our understanding of the complex relationships within student groups at a commuter college. The findings showed that having multiplex ties helps students thrive socially and academically (McCabe, 2016). For this reason, it is important to understand how students find support in each other when they are striving to orient themselves in higher education, whether in online or face-to-face learning environments. The case presented is also an example how a mixed-methods social network study can provide pedagogical insights that are valuable when designing a study program.

One overarching theme in the students’ accounts of their study-related networks is how the networks help them remain engaged in their studies; this is in line with previous research (e.g., Thomas, 2012). Students described how the different relationships provided different kinds of support, all of which built engagement in different ways. They reported that the friendship network made coming to school fun and provided access to information, both of which are important to academic achievement (e.g., Hommes et al., 2012). In line with McCabe’s (2016) findings, students indicated that the multiplex relationships were central to their academic success, as such relationships provided both task-related and emotional support (e.g., Rienties & Tempelaar, 2018). Students in the same network were “in the same boat,” “understood each other,” and having their support contributed the most to learning and engagement.

The interviewed students had a small number of multiplex relations, 1–2 peers whom they trusted and could rely on in their studies; this pattern is consistent with that found in the cohort as a whole (Author, 2021). Previous research has shown that commuter students have a smaller number of friendship and multiplex relations (e.g., Author et al., 2021) than students who live on or close to campus. There is inconclusive evidence regarding the importance of the working network to academic outcomes, whereas research has provided consistent evidence showing the importance of the friendship network to successful academic outcomes (e.g., Hommes et al., 2012; Thomas, 2012; Tomás-Miquel et al., 2016). In the present cohort, there were no significant differences in network size between Swedish commuter and non-commuter students, whereas immigrant commuter students had fewer friendship and working relations than did immigrant students who did not commute. In the cohort, immigrant commuter students earned fewer credits in nominal time than did Swedish commuter students (REF: Author, 2021).

Embeddedness in a larger friendship group is important, as students then have access to a larger pool of relationships for information, meaning there are more people with whom they can potentially develop multiplex relationships. Students with limited friendship relations both lack access to information and have limited opportunities to form multiplex relationships. This means that they are potentially locked into their limited network early in the program. A sense of regret could be detected in interviews with the students with limited networks: Taylor, Monawar and Ahmed. According to Taylor”new *gangs were created, and I was outside*,” and for Monawar, “*the second year was difficult*” because he knew only one other student in the class. Taylor struggled and was unsure of the relevance of getting an education, and both Ahmed and Monawar dropped out during the second year. This shows how having a very limited multiplex network and few friendship relations may ultimately impact academic achievement and students’ epistemological development. Intragroup (friendship) relations, provide access to expertise and critical reflection, which have been positively associated with academic performance (Gašević et al., 2013) and creativity (Tomás-Miquel et al., 2016).

One finding from the study is important for the program in question: This is the fact that the very factors that enabled participation for many students were perceived as barriers by other, less well-integrated students. The research has revealed various barriers to participation, such as institutional, situational, and dispositional barriers (e.g., Goto & Martin, 2009). One institutional barrier all interviewed students mentioned was orientation week, which excluded shy students or immigrant students who chose not to participate for religious or cultural reasons. Shyness and lack of social and study skills were dispositional barriers mentioned by both Swedish and immigrant students. Old friends from high school and commuting were situational barriers, mainly for immigrant students, who preferred to study with other immigrant students in their hometown. One implication for the program in question is that orientation week, which was important for relationship building among Swedish students, may need to be modified. The organization of activities cannot be left to the student union alone, but faculty need to work strategically to make the student union orientation week activities more inclusive, and in this specific context, less focused on partying and alcohol consumption. One immigrant student had thought about it and suggested: “*It’s not for us /…/ why not play football, we all love football*.”

The present results offer a complex picture of commuter students and of commuting as a barrier to participation. Previous literature has pointed out that commuter students are less likely to fully engage with their peers (e.g., Pokorny et al., 2017) or to participate in campus activities after class (Biddix, 2015). Some of the commuter students had limited networks and some struggled academically, but the commute was generally not seen as something negative. Some Swedish commuter students in the study, such as Ed and Mary, had just as many relationships, and Swedish commuter students succeeded in their studies to the same extent as their non-commuting counterparts (Author, 2020, 2021). This is in line with Gianoutsos and Rosser (2014), who found no difference in retention and academic standing between commuters and non-commuters.

In contrast, the commute was seen as a barrier mainly for the immigrant students, whose decision not to participate in full may have depended on several overlapping barriers. This finding is corroborated by previous studies of the same cohort regarding background factors and self-assessed preparedness, which have shown that immigrant students who commuted longer also earned fewer credits (e.g., Author, 2021; London Higher, 2019). Thus, commuting created an extra barrier primarily for immigrant students, who also mentioned having difficulties forming academically supportive relationships. The problem could partly be linked to a wider integration problem detected in this specific program, which is may well be similar to the situation for programs in other higher education contexts. Students talked about *commuters* and *non-commuters*, and how it was difficult to cooperate. Sometimes they added “apart from X, who is one of us.” In many cases, what they described was a divide based on ethnicity, rather than on commuting.

The quantitative analysis of the network data confirmed this view, indicating that most of the multiplex networks were homophilous regarding gender and ethnicity, revealing a division between ethnic Swedes and students with an immigrant background (see Author, 2021). In the interviews, Swedish students felt immigrant commuters preferred staying in their hometown to study and did not want to take part in social activities outside the classroom, such as orientation week, corroborating the picture of commuter students in previous literature (e.g., Pokorny et al., 2017). The immigrant commuter students also partly confirmed this view; they preferred studying in their hometowns with other commuter students but went to campus when necessary. They further explained how they formed multiplex relationships mainly with other immigrant students who lived in the same town. Students talked about how they, for example, were “the same age” or had the same “background,” referring to how they had grown up in the same place. There is consistent evidence showing that students form homophilous networks, that is, they prefer forming relationships with peers similar to themselves regarding, for example, gender, race/ethnicity, socioeconomic background or age, and cultural preferences (e.g., Rienties & Tempelaar, 2018; Rienties et al., 2015) as well as academic performance (e.g., Gašević et al., 2013).

Students form relationships based on preference or opportunity (e.g., Hommes et al., 2012). One opportunity for forming relationships that students mentioned was group work during the first semester, which has previously been found to be a strong predictor of relationships that students develop into friendships, at least temporarily (Rienties & Nolan, 2014). Other meeting places mentioned were orientation week activities and the commute to school. The friendships that lasted and developed into constructive and long-lasting multiplex relationships were those that “worked out,” in the sense that students found both the emotional and instrumental support needed for their studies. They spoke of how they continued working together because they “worked in the same way” or had the “same level of ambition,” which is in line with how students tend to form relationships with other students who perform at the same level (e.g., Gašević et al., 2013). Students who chose not to participate in orientation week, or for whom group work during the first semester did not work out, remained on the periphery of the cohort. They eventually formed a few friendship and multiplex relations, as students are less open to forming new relationships after the first year (e.g., Mamas, 2018).

A strong tendency toward homophily in student networks could have negative effects on students’ knowledge development. According to Curşeu and Pluut (2013), less diversity in groups leads to fewer chances for students to learn from or be motivated by each other. Students benefit from diversity, as it results in greater complexity of the collective knowledge in the group. Students also benefit from working with more motivated peers who contribute drive and organization. At the same time, greater disparity has a negative effect on teamwork processes and interpersonal interaction. Students were aware of both the benefits of collaboration with others, as pointed out in other studies (e.g., Mamas, 2018), and the drawbacks, which is why they preferred working with their multiplex relationships, especially on high-stakes assignments; in this context, high-stakes mean the assignments were decisive for their course grade.

In sum, both strong multiplex and weaker friendship relations are important to academic outcomes (Rienties & Tempelaar, 2018), and the interviews clearly indicate that students who performed less well did not have the same access to valuable information that their better-connected peers did. These students also spoke with regret about not knowing many people and hardly knowing the names of people in the class. In the target program, students are strategically mixed during the first semester, but are thereafter either free to choose their own groups or directed to work with certain others, depending on the preference of the teacher in question. In the interviews, students reported preferring to decide for themselves, wanting to work with people they can trust. Arguably, it is important to let them do this, especially when the tasks are complex, and their course grade is at stake.

One possible suggestion for the program in question is to organize activities in and around the classroom that enable students to form stable and rewarding multiplex relationships. On the other hand, the program should develop a model for group work that gives students the choice to work with multiplex relationships to the extent possible, while also offering them ample opportunity to get input from other peers with a more diverse background. This could be achieved if faculty were to engage more strategically in group formation and group work throughout the program.

During the first year, it is important to assign students to groups, as this allows all students to form constructive strong ties and important weak ties within the cohort. Group work and group processes must then be closely monitored to mitigate negative stereotyping and team conflict (Curşeu & Pluut, 2013). The further the students come in their studies, gradually forming multiplex relationships, the more they should be able to self-select, especially for more complex group assignments or for their final bachelor’s thesis. At the same time, to mitigate the negative effects of homophily on knowledge development and creativity, and increase access to information, students should be given ample opportunities to work together on low-stakes assignments in class or to discuss questions related to their high-stakes assignments with representatives from other groups.

## Limitations

The present study has several limitations. First, it is based on interviews from 2016. The situation for today’s students is surely different, especially as teaching has now partly transitioned to being online. At the same time, current research on student networks has revealed the importance of student relationships (Felten & Lambert, 2020), especially overlapping, multiplex relationships (Elmer et al., 2020; REF). Second, it must be kept in mind that the generalizability of the present findings is limited, as this is only a case study involving a single round of data collection, and a limited number of participants. However, there might be valuable implications for other educational contexts. Like all in-depth interpretive studies, potential avenues are highlighted for comparable situations, such as distant learning universities and the effects of pandemic lockdowns.

A third limitation is that the sample of interviewed students is limited to students in their third year, thus the perspective of less successful dropouts is missing. It would be valuable to explore how these students experienced their multiplex relationships. Finally, students were not asked to indicate strength of tie in the initial survey, and this limitation in the data makes it difficult to fully explore the complex, multiplex relationships.

At the same time, the study offers a rare glimpse into students’ understanding of their relationships and networks, which may be valuable to faculty at both the program and module level in various educational settings. A discussion about how to strategically work with student group constellations to foster both weak and strong ties may lead to improvements in learning, retention, and integration.

## Data Availability

available upon request.

## Code Availability

available upon request.

## References

[CR1] Alfano H, Eduljee N (2013). Differences in work, levels of involvement, and academic performance between residential and commuter students. College Student Journal.

[CR2] Biancani S., & McFarland D. A. (2013) Social networks research in higher education. In: M. Paulsen (Eds.), *Higher Education: Handbook of Theory and Research* (vol 28). Springer. 10.1007/978-94-007-5836-0_4

[CR3] Biddix P (2015). Strategies for assessing commuter students. New Directions for Student Services.

[CR4] Borgatti, S. P., Everett, M. G., & Freeman, L. C. (2002). *Ucinet 6 for windows: Software for social network analysis*. Analytic Technologies.

[CR5] Braun V, Clarke V (2006). Using thematic analysis in psychology. Qualitative Research in Psychology.

[CR6] Brouwer J, Jansen E (2019). Beyond grades: Developing knowledge sharing in learning communities as a graduate attribute. Higher Education Research and Development.

[CR7] Chen B, Wang F, Song J (2012). Are they connected? Exploring academic and social networks among MPA students as a Chinese University. Journal of Public Affairs Education.

[CR8] Cohen, L., Manion, L., Morrison, K. (2007). *Research methods in education*. Routledge.

[CR9] Crouch M, McKenzie H (2006). The logic of small samples in interview-based qualitative research. Social Science Information.

[CR10] Curşeu PL, Pluut H (2013). Student groups as learning entities: The effect of group diversity and teamwork quality on groups' cognitive complexity. Studies in Higher Education.

[CR11] Elmer T, Mephan K, Stadtfeld C (2020). Students under lockdown: Comparisons of students’ social networks and mental health before and during the COVID-19 crisis in Switzerland. PLoS One.

[CR12] Esterhazy, R. (2019). Re-conceptualizing feedback through a sociocultural lens. In M. Henderson, R. Ajjawi, D. Boud, & E. Molloy (Eds.), *The impact of feedback in higher education. Improving assessment outcomes for learners*. Switzerland Palgrave Macmillan. 10.1007/978-3-030-25112-3_5

[CR13] Felten P, Lambert LM (2020). Relationship-rich education: How human connections drive success in college.

[CR14] Fjelkner, A. (2020). Business students’ perceptions of their readiness for higher education studies and its correlation to academic outcome. *Journal for Advancing Business Education*, *2*(1), 74–92.

[CR15] Fjelkner-Pihl, A. (2022). The constructive overlap: A study of multiplex ties in students’ study-related networks and academic performance. *Innovative Higher Education**47*, 273–295. 10.1007/s10755-021-09576-4 10.1007/s10755-021-09576-4 PMC8427153 34518733

[CR16] Gašević D, Zouaq A, Janzen R (2013). "Choose your classmates, your GPA is at stake!" The association of cross-class social ties and academic performance. American Behavioural Science.

[CR17] Gianoutsos D, Rosser V (2014). Is there still a considerable difference? Comparing residential and commuter student profile characteristics at a public, research, commuter university. College Student Journal.

[CR18] Goto ST, Martin C (2009). Psychology of success: Overcoming barriers to pursuing further education. The Journal of Continuing Higher Education.

[CR19] Grunspan DZ, Wiggins BL, Goodreau SM (2014). Understanding classrooms through social network analysis: A primer for social network analysis in education research. CBE-Life Science Education.

[CR20] Henderson M., Ajjawi R., Boud D., Molloy E. (2019) Identifying feedback that has impact. In: M. Henderson, R. Ajjawi, D. Boud, E. Molloy (Eds.), *The impact of feedback in higher education.* Palgrave Macmillan, Cham. 10.1007/978-3-030-25112-3_2

[CR21] Héliot, Y. F., Mittlemeier, J., & Rienties, B. (2020). Developing learning relationships in intercultural and multi-disciplinary environments: A mixed method investigation of management students’ experiences. *Studies in Higher Education,**45*(11), 2356–2370. 10.1080/03075079.2019.1610865

[CR22] Higher L (2019). Commuter Students in London: Results of a pilot project on factors affecting continuation.

[CR23] Hommes J, Onyebuchi AA, de Grave W, Schuvirth LW, Scherpbier AJ, Bos GM (2014). Medical students perceive better group learning processes when large classes are made to seem small. PLoS One.

[CR24] Hommes J, Rienties B, de Grave W, Bos G, Schuwirth L (2012). Visualising the invisible: A network approach to reveal the informal social side of student learning. Advanced Health Science Education.

[CR25] Mamas C (2018). Exploring peer relationships, friendships and group work dynamics in higher education: Applying social network analysis. Journal of Further and Higher Education.

[CR26] McCabe JM (2016). Connecting in College: How Friendship Networks Matter for Academic and Social Success.

[CR27] McPherson M, Smith-Lovin L, Cook JM (2001). Birds of a feather: Homophily in social networks. Annual Review of Sociology.

[CR28] Methot JR, Lepine JA, Podsakoff NP, Christian JS (2016). Are workplace friendships a mixed blessing? Exploring tradeoffs of multiplex relationships and their association with job performance. Personnel Psychology.

[CR29] Newbold JJ (2015). Lifestyle challenges for commuter students. New Directions for Student Services.

[CR30] Palinkas, L., Horwitz, S., Green, C., Wisdom, J., Duan, N., & Kimberly, H. (2015). Purposeful sampling for qualitative data collection and analysis in mixed method implementation research. *Administration and Policy in Mental Health and Mental Health Services Research,* 533–544. 10.1007/s10488-013-0528-y 10.1007/s10488-013-0528-y PMC4012002 24193818

[CR31] Patterson MB (2018). The forgotten 90%: Adult nonparticipation in education. Adult Education Quarterly.

[CR32] Pokorny H, Holley D, Kane S (2017). Commuting, transitions and belonging: The experiences of students living at home in their first year at university. Higher Education.

[CR33] Rienties B, Hernandez Nanclares N, Jindal-Snape D, Alcott P (2012). The role of cultural background on team division in developing social learning relations in the classroom. Journal of Studies in International Education.

[CR34] Rienties B, Johan N, Jindal-Snape D (2015). Bridge building potential in cross-cultural learning: A mixed method study. Asian Pacific Education Review.

[CR35] Rienties B, Nolan E-M (2014). Understanding friendship and learning networks of international and host students using longitudinal Social Network Analysis. International Journal of Intercultural Relations.

[CR36] Rienties B, Tempelaar D (2018). Turning groups inside out: A social network perspective. Journal of the Learning Sciences.

[CR37] Swedish Higher Education Authority. (2019). *Utländsk bakgrund (%) bland högskolanybörjare*. https://www.uka.se/statistik. Accessed 20 Oct 2021.

[CR38] Thomas L (2012). What works? Student retention & success. Building student engagement and belonging at a time of change: Final report from the What works? Student retention and success programme.

[CR39] Thomas L (2019). Qualitative perceptions of students about commuting and studying in London.

[CR40] Tomás-Miquel J-V, Expósito-Langa M, Nicolau-Juliá D (2016). The influence of relationship networks on academic performance in higher education: A comparative study between students of a creative and a non-creative discipline. The International Journal of Higher Education Research.

[CR41] Wang, X., Hegde, S., Son, C., Keller, B., Smith, A., & Sasangohar, F. (2020/21). Investigating mental health of US college students during the COVID-19 pandemic: Cross-sectional survey study. *Journal of Medical Internet Research*, *22*(9), e22817. 10.2196/22817 10.2196/22817 PMC7505693 32897868

[CR42] Warfvinge, P., Löfgreen, J., Andersson, K., Roxå, T., & Åkerman, C. (2021). The rapid transition from campus to online teaching–how are students’ perception of learning experiences affected? *European Journal of Engineering Education*, 1–19. 10.1080/03043797.2021.1942794

[CR43] Wilcox P, Winn S, Fyvie-Gauld M (2005). 'It was nothing to do with the university, it was just the people': The role of social support in the first-year experience of higher education. Studies in Higher Education.

